# Measles and Varicella Vaccination Program in a Hospital: Implementation and Impact on Contact Tracing

**DOI:** 10.3390/vaccines11071131

**Published:** 2023-06-21

**Authors:** Alicia Siew Ting Loi, Revathi Sridhar, See Ming Lim

**Affiliations:** 1 Well Programme, Alexandra Hospital, Singapore 159964, Singapore; 2 Epidemiology Unit, Division of Infectious Diseases, National University Hospital, Singapore 119074, Singapore; revathi_sridhar@nuhs.edu.sg; 3 Occupational Health Clinic, National University Hospital, Singapore 119074, Singapore; see_ming_lim@nuhs.edu.sg

**Keywords:** measles vaccine, varicella vaccine, prevention and control, occupational health, immunization programs, health personnel, contact tracing

## Abstract

Healthcare workers are recommended to get vaccinated against measles and varicella. This study aims to describe the implementation process of a measles and varicella vaccination program and report on the reduction in the number of susceptible healthcare workers exposed to measles, varicella, and disseminated herpes zoster at a tertiary hospital. The Consolidated Framework for Implementation Research (CFIR) model was used to describe the vaccination program implemented from October 2015 to September 2018. The number of exposed, reviewed, and susceptible healthcare workers during contact tracing for exposure to (a) measles and (b) varicella and disseminated herpes zoster in 2016, 2017, and 2018 is reported. A total of 6770 (95%) out of 7083 healthcare workers completed their immunization review by 2018. In 2016, 20 (10%) out of 198 healthcare workers exposed to measles were considered susceptible. In 2018, no one was found susceptible out of the 51 staff members exposed to measles (*p* < 0.01). For exposure to varicella and disseminated herpes zoster, seven (5%) out of 154 exposed healthcare workers were susceptible in 2016. In comparison, only two (1%) out of 377 exposed healthcare workers in 2018 were susceptible (*p* < 0.01). The vaccination program effectively reduced number of healthcare workers susceptible to measles, varicella, and disseminated zoster.

## 1. Introduction

Healthcare workers are exposed to communicable diseases during the course of their work and are at risk of acquiring occupational infections. The Centers for Disease Control and Prevention (CDC) recommends healthcare workers to be vaccinated against a series of infectious diseases such as hepatitis B, measles, mumps, and rubella (MMR), varicella, pertussis, and meningococcal disease [1].

Measles and varicella are highly contagious and can spread by respiratory aerosols [2,3]. In a hospital, cases of measles and varicella infection will require airborne precaution as part of infection control measures. Similarly, cases of disseminated herpes zoster infection caused by reactivation of the varicella-zoster virus (VZV) will also need airborne precaution [4].

Infection prevention measures for healthcare workers exposed to the measles virus and VZV at work including contact tracing and medical management are essential to prevent and control the spread of disease in a hospital setting [5,6]. However, these processes are time-consuming, resource-intensive, and disrupts healthcare operations, especially if susceptible healthcare workers are required to take a leave of absence to prevent the spread of infection [7,8].

Measles and varicella infection are highly preventable with vaccination [6,9,10,11,12]. Increased vaccination coverage against measles virus and VZV will protect healthcare workers from contracting occupational infections.

An internal audit conducted in a tertiary hospital in Singapore found most of its staff lacking in the documentation of immunity status against vaccine-preventable diseases such as measles and varicella. The Ministry of Health in Singapore recommends healthcare workers to be vaccinated against MMR, varicella, influenza, hepatitis B, tetanus, diphtheria, and pertussis (Tdap) [13].

We aim to describe the implementation process of a measles and varicella vaccination program and report on the reduction in the number of susceptible healthcare workers exposed to measles, varicella, and disseminated zoster infection at the hospital. Vaccination against measles is performed as part of MMR vaccination. Although vaccination against measles and varicella for healthcare workers is widely advocated, a detailed description of the implementation process and direct outcomes during epidemiological investigation of exposed healthcare workers has not been widely reported.

## 2. Materials and Methods

A hospital-wide vaccination program, which includes vaccination against measles and varicella, was first planned in May 2015, in line with the recommendation from the Ministry of Health, Singapore [13]. We used the Consolidated Framework for Implementation Research (CFIR) model to describe the planning and implementation of the vaccination program [14]. CFIR consists of five major constructs: intervention characteristics, outer setting, inner setting, characteristics of the individuals involved, and the implementation process [15].

The vaccination program for healthcare workers was implemented at the hospital in Singapore from October 2015 to September 2018.

Intervention characteristics: the vaccination program was an initiative from the hospital to protect its staff and patients against nosocomial infections. The program was introduced and communicated to the head of departments and nursing leaders via physical meetings and e-mails. The benefits of having documented immunity to measles and VZV were outlined to the relevant stakeholders to ensure support from all parties. A timeline for the completion of the project was set to three years. All costs were covered by the hospital. The heads of department then disseminated the vaccination program information to their team members.

Outer setting: the vaccination program ensured that the healthcare workers were vaccinated against vaccine-preventable diseases, in line with the vaccination recommendations from the Ministry of Health in Singapore.

Inner setting: the hospital was a large 1200-bedded tertiary hospital, with a staff strength of approximately 7000 workers. It also functioned as a teaching hospital, with over fifty medical, surgical, and dental specialties. All staff without immunization records were required to undergo a medical review under the vaccination program.

Characteristics of the individuals involved: all healthcare workers including physicians, nurses, allied health members, administrators, and housekeeping staff were targeted in the program. The healthcare workers may be employed by the hospital or external partners.

**Figure 1:**
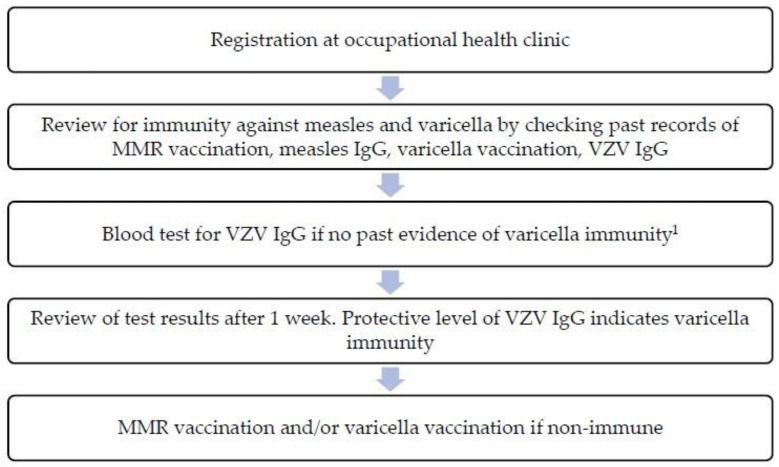
Workflow of varicella and MMR vaccination program. ^1^ IgG serology for measles, mumps, and rubella IgG may be performed if MMR vaccine is contraindicated for the healthcare worker.

Implementation process: the vaccination review process was carried out by the hospital’s occupational health physician and nurses at the occupational health clinic (Figure 1). Healthcare workers taking care of susceptible patients (e.g., patients at oncology wards, pediatric wards) and staff working at areas with high infectious disease exposure risk (e.g., Emergency Department) were given priority with earlier review sessions. For immunity against measles, healthcare workers were required to have a record of two MMR vaccine doses administered at least 28 days apart or protective level of measles immunoglobulin G (IgG) (laboratory reference ≥250 mIU/mL). Those without proof of MMR immunization were vaccinated. For immunity against varicella, healthcare workers were required to have a record of two doses of varicella-zoster vaccine or protective level of VZV IgG (laboratory reference ≥100 mIU/mL). Healthcare workers without previous varicella-zoster vaccination and a negative VZV IgG were vaccinated. Serum IgG antibodies against measles and varicella were measured using enzyme-linked immunosorbent assay (ELISA). All serology test results and records of vaccination were uploaded into the hospital’s electronic health records.

By the end of 2016, 3516 (58%) out of 7076 staff had completed their immunization review. By the end of the vaccination program in 2018, a total of 6770 (95%) out of 7083 healthcare workers had completed their vaccination review.

Cases of measles, varicella, and disseminated zoster in the hospital were reported to the hospital’s Epidemiology Unit for initiation of the contact tracing process. Verification of immunization status for healthcare workers with high-risk exposure was conducted by checking the electronic medical records. Healthcare workers with complete vaccination records or with previous protective IgG levels for the respective disease of exposure were considered immune. Healthcare workers without documented proof of immunity had to attend an urgent medical evaluation at the occupational health clinic to assess immunity against the disease of exposure. Reviewed staff without proof of vaccination underwent serological testing for VZV IgG or measles IgG for exposure to varicella, disseminated herpes zoster, or measles respectively. If the corresponding IgG result was negative, the worker was considered susceptible and was put on leave from work during the incubation period of the disease.

We report on the number of exposed, assessed (at the occupational health clinic), and susceptible healthcare workers for exposure to (a) measles cases and (b) varicella and disseminated herpes zoster infection cases for the years 2016, 2017, and 2018. The proportion of healthcare workers reviewed at the occupational health clinic and susceptible healthcare workers was reported as an outcome of the vaccination program. We used Stata 17 to perform a descriptive analysis of the outcome.

This study was approved by the National Healthcare Group, Domain-Specific Review Board, for exemption of a formal review.

## 3. Results

The number of healthcare workers assessed at the occupational health clinic for exposure to measles, varicella, and disseminated herpes zoster and the number of susceptible healthcare workers were reduced throughout the vaccination program.

In 2016, a total of 198 healthcare workers were exposed to measles cases (Table 1). Out of the 198 staff members, 101 (51%) workers did not have documentation of measles immunity (two doses of MMR vaccine or previous protective level of measles IgG) and were assessed at the occupational health clinic. Twenty (10%) workers were not able to demonstrate past records of MMR vaccination and had titers of measles IgG below the protective level. These 20 workers were considered susceptible and had to be put on medical leave for several days to prevent the spread of nosocomial infection. In comparison, five (10%) out of 51 exposed healthcare workers were assessed at the occupational health clinic in 2018. No one was found susceptible to measles (*p* < 0.01).

With respect to varicella and disseminated zoster, a total of 154 healthcare workers were exposed in 2016 (Table 2). Out of the 154 exposed staff members, 114 (74%) workers did not have documentation of varicella immunity (two doses of VZV vaccine or previous protective level of VZV IgG) and were assessed at the occupational health clinic. Seven (5%) workers were not able to demonstrate past records of VZV vaccination and had titers of VZV IgG below the protective level. These seven workers were considered susceptible and were put on medical leave. In comparison, 17 (5%) out of 377 exposed healthcare workers were assessed at the occupational health clinic in 2018. Only two (1%) were considered susceptible (*p* < 0.01).

## 4. Discussion

The vaccination program took approximately three years to complete, and successfully ensured immunity against measles and VZV for most of the healthcare workers. Based on CDC recommendations, healthcare workers were considered immune to measles after two doses of the MMR vaccine (administered at least 28 days apart) or if they had measles IgG [1]. Similarly, healthcare workers were considered immune to VZV if they received two doses of the VZV vaccine or were able to show laboratory evidence of immunity [1]. Commercial VZV IgG ELISAs are used for screening of VZV immunity. Both VZV-specific antibodies and VZV-specific T cells generated after primary VZV infection or vaccination confer immunity. While VZV IgG prevents primary VZV infection upon exogenous re-exposure of the virus, VZV-specific T cells limits severity, prevents reactivation, and aids in recovery of the disease [16,17,18].

The high vaccination uptake rate can be attributed to several intrinsic and extrinsic factors. During the implementation phase of the program, measures were taken to address potential challenges such as vaccine hesitancy and the requirement for additional manpower and laboratory resources.

At a personal level, all staff was individually counseled by an occupational health doctor on the indications of vaccination against MMR and varicella. The personalized one-on-one interaction between the occupational health doctor and staff was identified as an important factor to reduce potential hesitancy towards vaccination [19,20,21]. Strong emphasis was placed on the benefits, effectiveness, and safety profile of the vaccines since knowledge on the benefits and safety of a vaccine was important to increase vaccination uptake [22,23,24,25]. The healthcare workers were generally receptive to MMR and VZV vaccination. A personal sense of professional responsibility among the healthcare workers towards their patients could have also contributed to the high vaccination uptake [21,22,25,26].

Information about the vaccination program was disseminated to the staff from the head of the department and senior colleagues in the same department. Support from senior colleagues and peers can positively influence the healthcare workers to undergo vaccination [27,28].

The vaccination program was supported by the hospital’s management, as part of infection prevention measures for both staff and patients. The cost of serological tests and vaccines were borne by the hospital, eliminating financial burden as a potential barrier to vaccination. With the support of the hospital’s management, additional manpower was assigned to assist the vaccination program as required.

Departments were allocated staggered immunization review timings to ensure a smooth vaccination process without significant disruption to the clinical operations. The arrangement with different departments increased vaccine accessibility to the staff who otherwise might encounter difficulty finding additional time in their schedule to attend the vaccination session. A well-organized hospital-based vaccination program can encourage vaccination uptake among its staff [28,29].

The vaccination program was also given an adequate timeline for completion, to prevent overloading of cases seen at the occupational health clinic and overloading of serological testing at the laboratory. The systematic approach utilized ensured a high immunity coverage against the measles virus and VZV within an acceptable period, without needing a large number of additional manpower to complete the program. Priority was given to healthcare workers taking care of susceptible patients (e.g., patients at oncology wards, pediatric wards) and staff working in areas with high infectious disease exposure risk.

The benefits of vaccination against the measles virus and VZV have been extensively published and agreed upon. Prevention of nosocomial disease transmission among healthcare workers and patients remains the primary objective of vaccination amongst healthcare workers [30]. Vaccination against vaccine-preventable diseases will also reduce perceived susceptibility and provide mental assurance among healthcare workers [21]. Vaccination prevents large-scale outbreaks by reducing the number of nosocomial infections transmitted from infected healthcare workers [9]. The wards in our hospital can accommodate up to eight beds per room. An infectious disease outbreak such as measles or varicella in wards with multiple beds in a room can be a major event, with numerous patients and healthcare workers potentially exposed to the disease. Investigation and control of a large infectious disease outbreak in a hospital are costly and result in many work-hours lost [5,7,31].

Following the increase in vaccination coverage, the proportion of healthcare workers assessed at the occupational health clinic and the proportion of susceptible healthcare workers exposed to measles, varicella, and disseminated zoster significantly fell. The hospital’s Epidemiology Unit was able to quickly verify the immunity status of exposed staff during the contact tracing process due to the updated immunization records. Fewer healthcare workers without immunization status were reviewed at the occupational health clinic, resulting in a reduction of manpower resources, cost, and time spent identifying and assessing the affected staff. Each medical evaluation session for a healthcare worker exposed to measles, varicella, or disseminated herpes zoster takes approximately 30 min at the occupational health clinic.

Disruption to operations will be minimized and substantial time savings can be gained as a result of high vaccination coverage. In a department with multiple exposed staff members, a high immunity level will ensure that the healthcare workers are not susceptible and be put on medical leave during the incubation period of the disease. A cost-benefit analysis may be useful to demonstrate the resource savings in the long-term period.

## 5. Conclusions

Vaccination against vaccine-preventable diseases such as varicella and measles are beneficial for healthcare workers. A suitable implementation framework, such as the CFIR may be utilized to review various aspects of a vaccination program. In a healthcare setting without significant disease outbreaks, a vaccination program against varicella and measles can be paced, with prioritization for staff working with susceptible patients and staff with higher exposure risk.

Updated immunization records of healthcare workers will enable efficient epidemiological investigation and management of healthcare workers exposed to measles and varicella infections. Fewer healthcare workers will be required to undergo urgent immunity assessment following exposure to measles or varicella, and the number of susceptible healthcare workers will be significantly reduced. In addition to reducing the risk of infection to healthcare workers and patients under their care, disruption to operations will be minimized and substantial resource savings may be gained in the long run.

## Figures and Tables

**Table 1:** Exposure to Measles.

Year	2016	2017	2018
Total exposed HCW ^1^, n	198	49	51
HCW assessed at OHC ^2^, n (%)	101 (51%)	10 (20%)	5 (10%)
Susceptible HCW, n (%)	20 (10%)	3 (6%)	0 (0%)

^1^ HCW = healthcare workers; ^2^ OHC = occupational health clinic.

**Table 2:** Exposure to Varicella and Disseminated Herpes Zoster.

Year	2016	2017	2018
Total exposed HCW ^1^, n	154	568	377
HCW assessed at OHC ^2^, n (%)	114 (74%)	69 (12%)	17 (5%)
Susceptible HCW, n (%)	7 (5%)	14 (2%)	2 (1%)

^1^ HCW = healthcare workers; ^2^ OHC = occupational health clinic.

## Data Availability

The data are not publicly available due to institutional restrictions.
